# Multi-Tissue Time-Domain NMR Metabolomics Investigation of Time-Restricted Feeding in Male and Female Nile Grass Rats

**DOI:** 10.3390/metabo12070657

**Published:** 2022-07-16

**Authors:** Hayden Johnson, Thomas Yates, Gary Leedom, Chidambaram Ramanathan, Melissa Puppa, Marie van der Merwe, Aaryani Tipirneni-Sajja

**Affiliations:** 1 Department of Biomedical Engineering, University of Memphis, Memphis, TN 38152, USA; htjhnson@memphis.edu (H.J.); tcyates1@memphis.edu (T.Y.); galeedom@memphis.edu (G.L.); 2 College of Health Sciences, University of Memphis, Memphis, TN 38152, USA; rchdmbrm@memphis.edu (C.R.); mpuppa@memphis.edu (M.P.); mvndrmrw@memphis.edu (M.v.d.M.)

**Keywords:** NMR, dietary metabolomics, high-fat diet, time-restricted feeding, metabolic syndrome, Nile grass rats, time-domain NMR

## Abstract

Metabolic disease resulting from overnutrition is prevalent and rapidly increasing in incidence in modern society. Time restricted feeding (TRF) dietary regimens have recently shown promise in attenuating some of the negative metabolic effects associated with chronic nutrient stress. The purpose of this study is to utilize a multi-tissue metabolomics approach using nuclear magnetic resonance (NMR) spectroscopy to investigate TRF and sex-specific effects of high-fat diet in a diurnal Nile grass rat model. Animals followed a six-week dietary protocol on one of four diets: chow ad libitum, high-fat ad libitum (HF-AD), high-fat early TRF (HF-AM), or high-fat late TRF (HF-PM), and their liver, heart, and white adipose tissues were harvested at the end of the study and were analyzed by NMR. Time-domain complete reduction to amplitude–frequency table (CRAFT) was used to semi-automate and systematically quantify metabolites in liver, heart, and adipose tissues while minimizing operator bias. Metabolite profiling and statistical analysis revealed lipid remodeling in all three tissues and ectopic accumulation of cardiac and hepatic lipids for HF-AD feeding compared to a standard chow diet. Animals on TRF high-fat diet had lower lipid levels in the heart and liver compared to the ad libitum group; however, no significant differences were noted for adipose tissue. Regardless of diet, females exhibited greater amounts of hepatic lipids compared to males, while no consistent differences were shown in adipose and heart. In conclusion, this study demonstrates the feasibility of performing systematic and time-efficient multi-tissue NMR metabolomics to elucidate metabolites involved in the crosstalk between different metabolic tissues and provides a more holistic approach to better understand the etiology of metabolic disease and the effects of TRF on metabolic profiles.

## 1. Introduction

Metabolic syndrome (MetS), a cluster of interrelated metabolic abnormalities characterized by obesity, insulin resistance, hypertension, and hyperlipidemia, has been increasing in prevalence, with ~35% of the United States population affected as of 2016, leading to its status today as a global epidemic [1,2,3]. MetS is a collection of conditions with no singular cause and is affected by genetics and environmental factors including sedentary lifestyle, physical inactivity, and dietary habits [4,5]. The epidemic of MetS is unlikely to be reduced without societal lifestyle changes [2], and some diet regimens have proven effective to reduce risk factors of metabolic disease [6]. Time-restricted feeding (TRF) is one such diet regimen in which food intake is limited to a set window of time each day, with fasting for the remaining time [7]. TRF has been increasing in popularity and has a relatively high compliance rate as there are no restrictions regarding what foods may be eaten, and food intake is ad libitum during the set window, unlike traditional fasting where caloric intake is restricted [8,9,10].

Circadian rhythm is an important regulator of metabolism in mammals that results in daily temporal oscillations in gene expression. Many genes fundamental to metabolism are expressed in the liver, and food intake is an important driver of their rhythmic oscillations in expression [11,12]. Prolonged and erratic daily eating periods are common in the United States and are associated with chronic metabolic disorder [13,14]. Irregular meal timing negatively impacts circadian metabolism, resulting in attenuated gene expression and metabolic inefficiency, whereas TRF seeks to align timing of daily food intake to improve metabolic function [13,15]. Previous studies suggest that TRF can be an effective strategy for weight loss and prevention of common chronic metabolic diseases such as obesity and type 2 diabetes [13,15].

MetS disrupts whole-body energy metabolism and affects multiple metabolically active tissues. In MetS, lipid regulation in white adipose tissue is disturbed, and excess energy intake triggers triglyceride accumulation in non-adipose organs such as the liver, heart, and skeletal muscle. It is important to understand the relationship between diet and tissue-level metabolomics, as there exists a great deal of crosstalk between metabolically active tissues that regulates the progression of diet-induced obesity into MetS [16,17,18,19,20,21]. Many dietary metabolomics studies focus on biofluids such as serum and urine for investigating metabolite changes, as they are easily accessible [22,23,24]. However, a previous study demonstrated that measurement of liver metabolites better reflects the metabolic effects of nutritional challenge compared to serum metabolites [25]. Profiling multiple metabolic organs can provide a more holistic approach and elucidate metabolites and biochemical pathways implicated in leading to MetS in this crosstalk. Only a limited number of studies to date have taken a tissue-level metabolomics approach to understanding TRF [25,26], but none of them performed multi-tissue metabolomics to understand the tissue crosstalk in MetS and the attenuating effects of TRF in multiple metabolic organs.

Nuclear magnetic resonance (NMR) spectroscopy is one of the primary analytical techniques used in metabolomics, along with mass spectrometry. Although mass spectrometry has a lower detection limit and can detect more metabolites, NMR has distinct advantages with its inherent nondestructive and quantitative nature, high reproducibility, relatively simple sample preparation, and the ability to detect and quantify numerous metabolites in a single NMR spectrum, hence making it an excellent high-throughput technique [27]. Further, isotopic labels are less complex to implement in NMR experiments, and metabolites of interest may possibly be detected by high-field magnetic resonance imaging (MRI) for noninvasive diagnosis and monitoring of diseases [28,29]. NMR analysis can provide a wealth of information regarding nutrition and health status and has been used to investigate the content of food at a molecular level [30], the effects of dietary intake [22,24], and metabolic responses in disease states [22,24].

NMR-based metabolomics is conventionally performed in the frequency domain and requires extensive spectral preprocessing in the form of apodization, zero-filling, and phase and baseline correction, followed by quantification by either integration or peak fitting [29]. These steps are time- and labor-intensive for analyzing cohorts of metabolomics data and can introduce user bias and inter-operator variability in metabolite quantification [31]. In this study, we investigate time-domain NMR parameter estimation using the complete reduction to amplitude–frequency table (CRAFT) workflow [31,32,33], which uses Bayesian probability theory to provide reproducible and optimal parameter estimates [31,34]. CRAFT simplifies critical metabolite identification and quantification steps of frequency and amplitude determination by bypassing the drawbacks associated with conventional frequency-domain NMR and can increase accuracy and automation for multi-tissue metabolomics.

Hence, the purpose of this study is to use CRAFT time-domain NMR analysis for metabolic profiling of liver, heart, and white adipose tissues in order to better understand the sex-specific and tissue-level responses to high-fat ad libitum and TRF diet regimens. In this study, the TRF protocol consisted of a six-hour feeding window followed by 18 h of fasting. Both early and late TRF protocols are evaluated, where eating is limited to the first or last six hours of a 12 h light cycle, respectively. Most animal studies to date have only examined TRF outcomes in nocturnal animals using either males [26,35,36,37,38] or females [39,40] rather than both. The current study investigates TRF in both sexes, as males and females handle many major metabolic functions such as fat storage and energy homeostasis differently [41,42]. In this study, Nile grass rats (NGR) were selected as the model animal as they are diurnal and have circadian regulation that is similar to humans. They also develop the constellation of MetS symptoms solely through dietary manipulation (no genetic or chemical alterations) and mimic much of the disease progression observed in humans [43,44,45].

The functional outcomes of the animal cohort used in this study were previously reported, demonstrating that high-fat TRF protocols had significantly lower weight gain and food consumption compared to high-fat ad libitum feeding [46]. Further, early TRF resulted in improved hepatic clock gene expression, lower liver weight, and reduced fasting blood glucose levels compared to ad libitum eating. However, no significant differences were detected for fasting insulin or plasma triglyceride levels for TRF compared to ad libitum. Sex differences were also noted in the previous study, with females having heavier livers and males having heavier gonadal white adipose tissue [46]. Complementary to the published functional outcomes, the objectives of the current study are to systematically investigate multi-tissue metabolomics and identify sex and tissue-level metabolomic differences between high-fat ad libitum and TRF protocols to better understand the development of MetS.

## 2. Results

**Figure 1:**
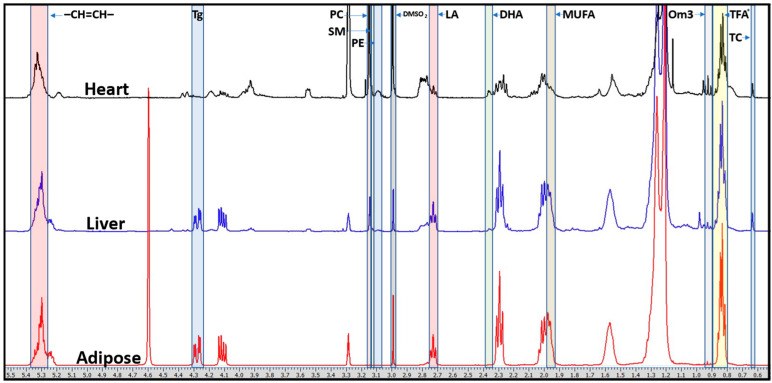
Representative cardiac, hepatic, and adipose lipid spectra obtained from a male Nile grass rat fed with a high-fat, time-restricted, evening feeding diet using proton nuclear magnetic resonance (^1^H-NMR) spectroscopy. Complete reduction to amplitude–frequency table (CRAFT) fingerprints are shown for targeted lipid resonances. Abbreviations: Tg, triglycerides; PC, phosphatidylcholine; SM, sphingomyelin; PE, phosphatidylethanolamine; DMSO_2_, dimethyl sulfone; LA, linoleic acid; DHA, docosahexaenoic acid; MUFA, monounsaturated fatty acids; Om3, omega-3 fatty acids; TFA (TFA* + Om3), total fatty acids; TC, total cholesterol; –CH=CH–, olefinic protons.

Chemical shift fingerprints for aqueous and lipid metabolites, respectively, are systematically applied for all tissue samples, with a representative lipid fingerprint shown in Figure 1. Summarized below are the dietary changes with respect to high-fat ad libitum (HF-AD), early TRF (HF-AM), late TRF (HF-PM), and sex differences observed in the metabolite concentrations for different tissues.

### 2.1. High-Fat Ad Libitum versus Chow

**Figure 2:**
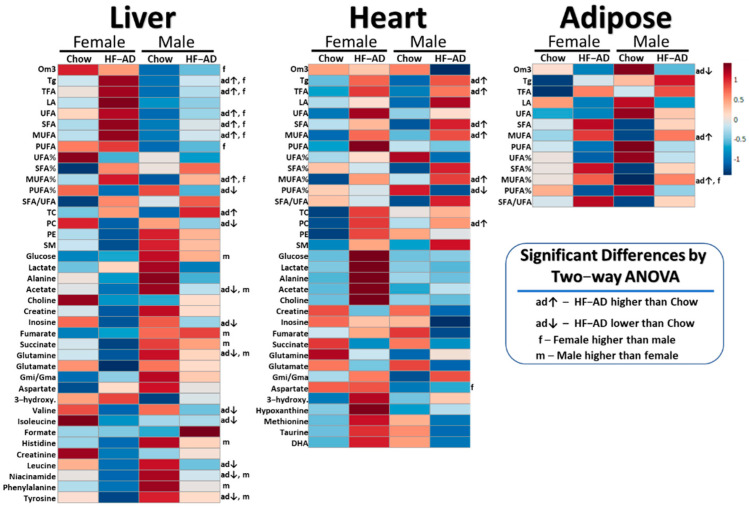
Heatmaps showing mean values of auto-scaled metabolites for both male and female chow and high-fat ad libitum (HF-AD) dietary groups in liver, heart, and adipose tissues of Nile grass rats (*n* =3–5 animals per group). The red and blue colors denote concentrations above and below the indicated metabolite mean concentration value, respectively, with darker colors indicating values farther from the mean. Significant main effects of diet and sex (*p* < 0.05) in metabolite concentrations from two-way ANOVA after post-hoc testing are also shown. Abbreviations: Tg = triglycerides; PC = phosphatidylcholine; SM = sphingomyelin; PE = phosphatidylethanolamine; LA = linoleic acid; DHA = docosahexaenoic acid; MUFA = monounsaturated fatty acids; PUFA = polyunsaturated fatty acids; UFA = unsaturated fatty acids; SFA = saturated fatty acids; Om3 = omega-3 fatty acids; TFA = total fatty acids; TC = total cholesterol; 3-hydroxy. = 3-hydroxybutyrate; Gmi/Gma = ratio of glutamine to glutamate.

Two-way ANOVA revealed changes induced by ad libitum high-fat feeding that led to statistically significant differences in some measurable metabolite concentration in the liver, heart, and adipose tissues compared to rats eating standard chow, as shown in Table 1 and Table 2 for lipids and aqueous metabolites, respectively. A heatmap summarizing ANOVA is displayed in Figure 2.

For all tissues, there were significant main effects of diet, with the HF-AD group having higher levels of monounsaturated fatty acids (MUFA) and MUFA% (the percentage of total fatty acids (TFA) that are MUFA) (*p* ≤ 0.016). The fatty acid composition of adipose tissue was further altered in HF-AD compared to chow, with the HF-AD rats exhibiting lower omega-3 concentrations (*p* = 0.028) and a close to significantly higher ratio of saturated fatty acids over unsaturated fatty acids (SFA/UFA) (*p* = 0.058). The liver and heart showed a greater degree of change in response to high-fat diet than adipose, with concentrations of hepatic and cardiac triglycerides, TFA, and SFA higher and the percentage of polyunsaturated fatty acids (PUFA%) lower in HF-AD rats (*p* ≤ 0.033). Main effects of diet were also noted for phosphatidylcholine, which was higher in the heart but lower in the liver for HF-AD compared to chow (*p* ≤ 0.031). Further significant changes noted for hepatic measures in HF-AD subjects were higher total cholesterol and unsaturated fatty acids (UFA) as well as lower levels of glutamine, inosine, isoleucine, leucine, niacinamide, tyrosine, and valine compared to chow (*p* ≤ 0.047).

**Figure 3:**
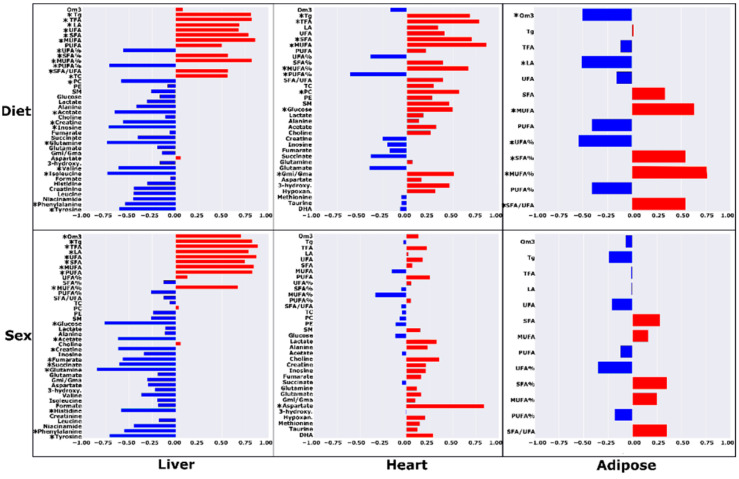
Correlations between metabolite levels with diet and sex in high-fat ad libitum (HF-AD) and chow-fed dietary groups for different tissues. For dietary partial correlations, metabolites with positive correlation coefficients are higher in HF-AD, and metabolites with negative coefficients are lower in HF-AD compared to chow. For sex partial correlations, metabolites with positive correlation coefficients are higher in females, and vice versa for negative coefficients compared to males. The metabolites with significant partial correlations (*p* < 0.05) are denoted with asterisks (*). Abbreviations: Tg = triglycerides; PC = phosphatidylcholine; SM = sphingomyelin; PE = phosphatidylethanolamine; LA = linoleic acid; DHA = docosahexaenoic acid; MUFA = monounsaturated fatty acids; PUFA = polyunsaturated fatty acids; UFA = unsaturated fatty acids; SFA = saturated fatty acids; Om3 = omega-3 fatty acids; TFA = total fatty acids; TC = total cholesterol; 3-hydroxy. = 3-hydroxybutyrate; Hypoxan. = hyphoxanthine; Gmi/Gma = ratio of glutamine to glutamate.

The partial correlations between metabolite concentrations and metadata controlled for diet or sex are shown in Figure 3. In the liver, there were higher concentrations of MUFA, TFA, triglycerides, SFA, linoleic acid, UFA, MUFA%, SFA%, the ratio of SFA/UFA, and total cholesterol, and lower levels of phenylalanine, creatine, phosphatidylcholine, PUFA%, UFA%, tyrosine, valine, acetate, inosine, isoleucine, and glutamine that significantly correlated with the HF-AD diet (R ≥ |±0.545|; *p* ≤ 0.035). For cardiac metabolites, higher levels of MUFA, TFA, SFA, triglycerides, MUFA%, phosphatidylcholine, glucose, and the ratio of glutamine over glutamate (Gmi/Gma), and lower levels of PUFA% significantly correlated with the HF-AD group (R ≥ |±0.0.488|; *p* ≤ 0.047). In adipose samples, higher levels of MUFA%, MUFA, SFA%, and SFA/UFA and lower levels of UFA%, omega-3, and linoleic acid were significantly correlated with the HF-AD group (R ≥ |±0.509|; *p* ≤ 0.044).

### 2.2. High-Fat Ad Libitum Sex Differences

Concentrations of several metabolites were measured at significantly different levels between male and female rats, primarily in the liver. Main effects of sex were detected in two-way ANOVA for several metabolites (Figure 2). In females, hepatic lipids including omega-3, triglycerides, TFA, UFA, SFA, MUFA, PUFA, and MUFA% were higher (*p* ≤ 0.013). Furthermore, in females, sphingomyelin was significantly lower, along with several aqueous metabolites including glucose, acetate, fumarate, succinate, histidine, glutamine, niacinamide, phenylalanine, and tyrosine (*p* ≤ 0.042). Female rats also exhibited higher cardiac levels of aspartate compared to males (*p* = 0.003).

Metabolic differences between the sexes were noted by partial correlation analysis as well (Figure 3). Higher levels of TFA, UFA, MUFA, PUFA, SFA, omega-3, MUFA%, linoleic acid, and triglycerides, and lower levels of hepatic glutamine, glucose, tyrosine, acetate, creatine, succinate, histidine, fumarate, and phenylalanine were significantly associated with females (R ≥ |±0.555|; *p* ≤ 0.032). A higher concentration of cardiac aspartate was significantly correlated with female rats (R ≥ |±0.843|; *p* = 4.15 × 10^−5^).

### 2.3. High-Fat TRF versus Ad Libitum

**Figure 4:**
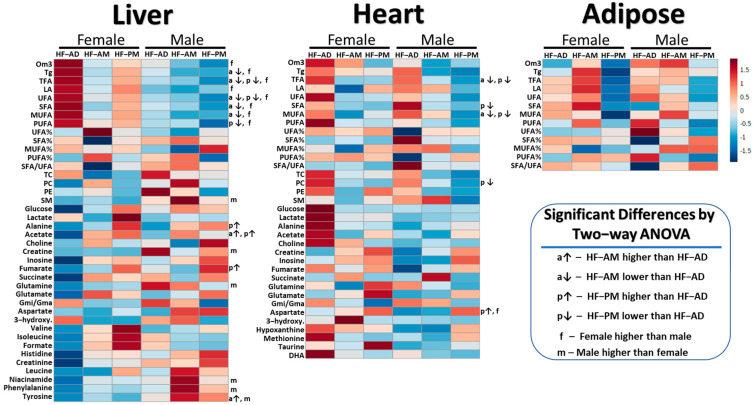
Heatmaps showing mean values of auto-scaled metabolites for both male and female high-fat ad libitum (HF-AD), high-fat morning time-restricted (HF-AM), and high-fat evening time-restricted (HF-PM) groups for different sexes in liver, heart, and adipose tissues of Nile grass rats (*n* = 3–5 animals per group). The red and blue colors denote concentrations above and below the indicated metabolite mean concentration value, respectively, with darker colors indicating values farther from the mean. Adipose lipids expressed no statistically significant differences in metabolite concentrations in response to TRF. Significant main effects of diet and sex (*p* < 0.05) in metabolite concentrations from two-way ANOVA after post-hoc testing are also shown. Abbreviations: Tg = triglycerides; PC = phosphatidylcholine; SM = sphingomyelin; PE = phosphatidylethanolamine; LA = linoleic acid; DHA = docosahexaenoic acid; MUFA = monounsaturated fatty acids; PUFA = polyunsaturated fatty acids; UFA = unsaturated fatty acids; SFA = saturated fatty acids; Om3 = omega-3 fatty acids; TFA = total fatty acids; TC = total cholesterol; 3-hydroxy. = 3-hydroxybutyrate; Gmi/Gma = ratio of glutamine to glutamate.

Regardless of the timing of the regimen, the main effects of diet were detected for the two TRF groups (AM, PM), which expressed lower concentrations of both cardiac and hepatic TFA compared to their ad libitum counterparts (Table 3 and Table 4, *p* ≤ 0.033). In TRF rats, hepatic UFA, triglycerides (AM only), SFA (AM only), MUFA (AM only), and PUFA (PM only) were significantly lower, and acetate, alanine (PM only), fumarate (PM higher than AM and AD), and tyrosine (AM only) were higher compared to HF-AD groups (*p* ≤ 0.041). For heart metabolites, phosphatidylcholine (PM only), SFA (PM only), and MUFA were significantly lower, and aspartate (PM higher than AM and AD) was significantly higher in the TRF groups compared to HF-AD (*p* ≤ 0.046). The heatmap in Figure 4 displays the TRF results of ANOVA for all tissues. In the adipose tissue, there were no statistically significant metabolic changes in response to TRF compared to HF-AD (Table 5).

Partial correlation analysis revealed several metabolites that were significantly correlated with diet in each of the TRF groups (Figure 3 and Figure 4). Lower concentrations of both cardiac and hepatic TFA and UFA were strongly associated (R ≥ |±0.482|; *p* ≤ 0.05) with both TRF regimens. Further, in the liver, TRF was strongly associated with significantly lower levels of Tg levels and higher acetate and alanine levels (R ≥ |±0.482|; *p* ≤ 0.05). In addition to the metabolites common to both TRF diets, certain metabolites were significantly associated with the AM group and not the PM regimen. Lower levels of hepatic total cholesterol, SFA, MUFA, and MUFA%, and higher levels of tyrosine, phenylalanine, and niacinamide were significantly correlated with the HF-AM diet (R ≥ |±0.515|; *p* ≤ 0.035). In heart, lower levels of MUFA, SFA, and phosphatidylethanolamine (PE) were associated with the HF-AM diet (R ≥ |±0.487|; *p* ≤ 0.048). For the HF-PM group, a lower level of hepatic PUFA and higher levels of fumarate, valine, inosine, histidine, and glucose were significantly correlated with the HF-PM diet (R ≥ |±0.521|; *p* ≤ 0.032). In heart, lower levels of MUFA, Gmi/Gma, PC, docosahexaenoic acid (DHA), and SFA, and higher levels of aspartate and glutamate were strongly associated with the HF-PM diet (R ≥ |±0.489|; *p* ≤ 0.046).

### 2.4. TRF Sex Differences

Main effects of sex determined by two-way ANOVA were similar to the chow/HF-AD comparison (Figure 4). In females, hepatic lipids including omega-3, triglycerides, TFA, UFA, SFA, MUFA, PUFA, and linoleic acid were higher (*p* ≤ 0.049). Furthermore, in females, sphingomyelin was significantly lower, along with several aqueous metabolites including creatine, glutamine, niacinamide, phenylalanine, and tyrosine (*p* ≤ 0.031). Female rats also exhibited higher cardiac levels of aspartate compared to males (*p* = 0.034).

**Figure 5:**
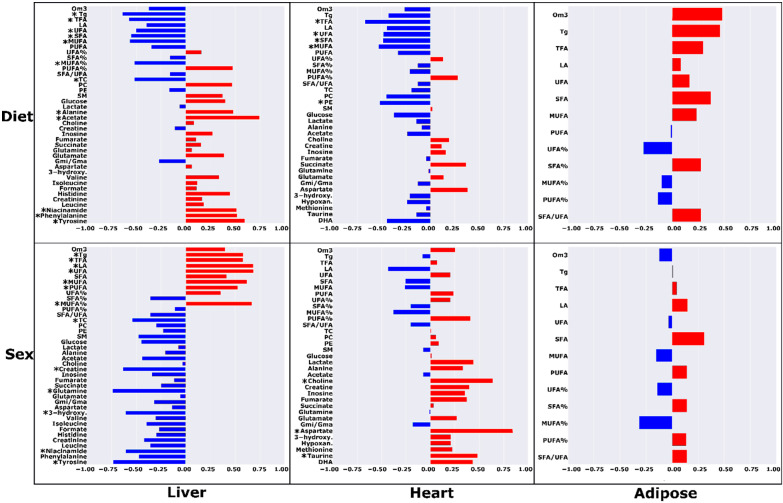
Correlations between metabolite concentration levels with diet and sex in high-fat ad libitum (HF-AD) and time-restricted morning (HF-AM) dietary groups. For dietary partial correlations, metabolites with negative coefficients are lower in HF-AM, and with positive correlation coefficients are higher compared to HF-AD. For sex partial correlations, metabolites with positive correlation coefficients are higher in females, and vice versa for negative coefficients compared to males. The metabolites with significant partial correlations (*p* < 0.05) are denoted with asterisks (*). Abbreviations: Tg = triglycerides; PC = phosphatidylcholine; SM = sphingomyelin; PE = phosphatidylethanolamine; LA = linoleic acid; DHA = docosahexaenoic acid; MUFA = monounsaturated fatty acids; PUFA = polyunsaturated fatty acids; UFA = unsaturated fatty acids; SFA = saturated fatty acids; Om3 = omega-3 fatty acids; TFA = total fatty acids; TC = total cholesterol; 3-hydroxy. = 3-hydroxybutyrate; Hypoxan. = hyphoxanthine; Gmi/Gma = ratio of glutamine to glutamate.

**Figure 6:**
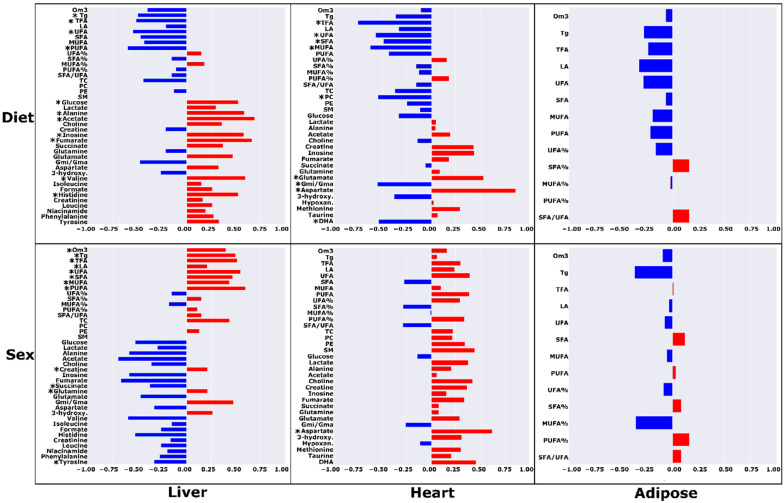
Correlations between metabolite concentration levels with diet and sex in high-fat ad libitum (HF-AD) and time-restricted evening (HF-PM) dietary groups. For dietary partial correlations, metabolites with negative coefficients are lower in HF-PM, and with positive correlation coefficients are higher compared to HF-AD. For sex partial correlations, metabolites with positive correlation coefficients are higher in females, and vice versa for negative coefficients compared to males. The metabolites with significant partial correlations (*p* < 0.05) are denoted with asterisks (*). Abbreviations: Tg = triglycerides; PC = phosphatidylcholine; SM = sphingomyelin; PE = phosphatidylethanolamine; LA = linoleic acid; DHA = docosahexaenoic acid; MUFA = monounsaturated fatty acids; PUFA = polyunsaturated fatty acids; UFA = unsaturated fatty acids; SFA = saturated fatty acids; Om3 = omega-3 fatty acids; TFA = total fatty acids; TC = total cholesterol; 3-hydroxy. = 3-hydroxybutyrate; Hypoxan. = hyphoxanthine; Gmi/Gma = ratio of glutamine to glutamate.

Metabolic differences between male and female animals were also noted by partial correlation analysis (Figure 5 and Figure 6) for the TRF AM and PM diets. Higher levels of TFA, UFA, MUFA, PUFA, linoleic acid, and triglycerides and lower levels of hepatic glutamine, tyrosine, and creatine were significantly correlated with females (R ≥ |±0.528|; *p* ≤ 0.029). A higher concentration of cardiac aspartate was significantly correlated with female rats (R ≥ |±0.621|; *p* ≤ 0.008).

## 3. Discussion

In this study, multi-tissue NMR metabolomics was performed using time-domain CRAFT to investigate high-fat feeding, TRF, and sex differences in metabolomic profiles in a diurnal model of MetS. Metabolite profiling and statistical analysis revealed that high-fat ad libitum feeding was associated with the accumulation of hepatic and cardiac triglycerides and fatty acids compared to standard Chow diet. Adipose tissue additionally underwent lipid remodeling, but without as extensive lipid accumulation as in liver and heart. TRF attenuated many of the measured effects of high-fat feeding, with both liver and heart having lower levels of fatty acids compared to ad libitum fed rats; however, no lipidomic changes were noted in the adipose tissue in response to TRF.

^1^H-NMR spectroscopy allowed for the measurement of 38, 33, and 12 metabolites in liver, heart, and adipose tissues respectively. Aqueous metabolites were not quantified in adipose tissues due to low natural abundance, and some metabolite peaks had relatively low signal-to-noise ratios (SNR) in some tissues that thus limited the total number of metabolites quantified in each tissue. Chemical shift fingerprints of aqueous and lipid metabolites were systematically applied for all tissue samples to quantify absolute concentrations of the metabolites. Time-domain processing using CRAFT bypassed user-dependent spectral preprocessing steps and allowed for semi-automated, time efficient quantification of targeted metabolites with an average processing time of ~5 min per sample.

### 3.1. Chow vs. HF-AD Diet Comparison

NMR metabolite profiling using CRAFT revealed several characteristic metabolic trends in response to ad libitum high-fat feeding compared to chow fed rats. Accumulation of fat in the liver and heart was evidenced by higher levels of triglycerides, MUFA, SFA, and TFA. Previous studies have shown that such ectopic accumulation of fat is considered lipotoxic and is associated with obesity and type 2 diabetes [16,47]. Further, studies in rats suggest increased lipid content and fatty acid availability in the heart are associated with impairment of cardiac function and increased oxidative stress [48]. In addition, the livers also had significantly higher total cholesterol, lower concentrations of phosphatidylcholine, greater levels of linoleic acid, and a higher SFA/UFA ratio that positively correlated with HF-AD compared to chow. Our lipid results in NGR are consistent with previous rat studies that demonstrated signs of hepatic steatosis with higher levels of cholesterol and triglycerides in male Wistar and Sprague–Dawley rats [49], and lower UFA-% and higher levels of fatty acids and triglycerides in Fischer rats [50] on high-fat feeding compared to standard chow diet. Further, levels of cardiac triglycerides were reported to be higher in male Fischer [51] and Wistar [52] rats when fed a high-fat diet compared to standard diet, similar to our results in NGR heart samples. In liver, aqueous metabolites showed lower concentrations of branched-chain amino acids (BCAAs) (leucine, isoleucine, and valine), aromatic amino acids (phenylalanine and tyrosine), acetate, inosine, glutamine, and creatine that were significantly associated with HF-AD compared to chow diet. These findings agree with a previous study that reported disruptions in BCAA and aromatic amino acid metabolism to be potential biomarkers of obesity and hepatic steatosis in humans [53]. Measures in adipose tissue revealed a change in relative rather than absolute amounts of fatty acids, with no significant change noted for triglycerides or TFA, a significantly higher SFA/UFA ratio, MUFA%, and SFA%, as well as lower PUFA%. The metabolite profiles in liver were more responsive to HF-AD feeding than those measured from heart samples, with 58% (23/40) of all measured hepatic metabolites significantly changed compared to 23% (8/35) of metabolites in heart tissue. The lipid profiles alone reveal all three metabolic tissues examined were susceptible to lipidomic remodeling under the HF-AD regimen, with 76% (13/17), 39% (7/18), and 54% (7/13) significantly altered lipid metabolite concentrations for liver, heart, and adipose, respectively, compared to chow diet.

### 3.2. TRF vs. HF-AD Diet Comparison

Restricting eating to a six-hour window each day was associated with several attenuating metabolic changes compared to those induced by the obesogenic HF-AD diet as noted above. Compared to HF-AD, rats on both TRF schedules had lower cardiac and hepatic TFA levels, which is evidence of less accumulation of ectopic lipids, and lower liver triglycerides, pointing to a lesser degree of steatosis [54]. These findings are consistent with the functional metabolic outcomes published in the first study of the same animal cohort that showed that TRF rats had lower weight gain, total fat percentage, and liver weights compared to the HF-AD group [46]. Our hepatic results are consistent with several recent studies in mice that report a reduction in hepatic steatosis and triglycerides in conjunction with TRF compared to ad libitum high-fat feeding. [26,39,55]. Similarly, a study in Wistar rats examined the effects of TRF on a high sucrose diet, and found TRF attenuated hepatic triglyceride accumulation [56]. Acetate levels were higher in both TRF groups (as seen in chow fed rats) compared to HF-AD group. These effects, which seemingly oppose the metabolic effects of high-fat feeding, agree with studies which suggest circadian meal alignment by TRF may be a beneficial dietary strategy for combatting metabolic disease [57,58].

Of the three tissues profiled, the liver was most responsive to TRF, with 30% and 28% of profiled metabolites significantly altered in HF-AM and HF-PM, respectively, when compared to the ad libitum group. Cardiac metabolites experienced less change, at 14% and 26% for AM and PM, respectively. No significant differences were noted in adipose lipids as a result of TRF. The sensitivity of hepatic metabolite levels and no notable changes in adipose lipid levels to TRF could be a result of the liver inherently experiencing greater intra- and inter-tissue circadian oscillations in metabolite levels, and thus responding more to circadian mealtime alignment. These findings are similar to a previous study that reported that the liver metabolome had many circadian oscillating metabolites compared to very few in white adipose tissue for both chow and high-fat dietary groups [57].

### 3.3. Sex-Dependent Differences

Comparing the metabolite profiles of females and males suggests a sexual dimorphism in hepatic metabolism. The livers of female rats had greater concentrations of triglycerides and fatty acids (TFA, UFA, SFA, MUFA, PUFA, and omega-3) and lower concentrations of glutamine, niacinamide, phenylalanine, and tyrosine. Interestingly, many of these metabolites also showed significant differences between the chow and HF-AD diets and between the HF-AD and TRF diets. The liver was the only tissue with such extensive sex-specific differences (48% of profiled hepatic measures significantly different between males and females for HF-AD compared to chow, and 43% and 30% of hepatic measures different for HF-AM and HF-PM, respectively, compared to HF-AD). These results agree with the functional metabolic outcomes previously reported for this study cohort that females had higher liver weights than males, indicating sexual differences in fat storage. Further, our findings were consistent with a previous study by Wells et al., which measured hepatic and adipose metabolites in C57BL/6J mice across five different diets and determined the impact of sex and diet was most evident in the liver [59]. No significant changes were noted for cardiac or adipose lipids between male and female rats in this study; however, the aspartate levels in the hearts were higher in females compared to male rats. The increased cardiac aspartate levels seen in females could potentially have antioxidant effects to protect against oxidative damage that might occur due to increased cardiac lipid accumulation [60,61].

### 3.4. Limitations

There are some notable study limitations. The current study is limited by the small sample size, with only 3–5 animals in each group by diet and sex (unequal number of rats/group due to some random deaths before the end of the study), which hinders a robust statistical evaluation of all significant metabolite changes. Multi-tissue metabolomics was performed on tissue samples collected at the end of the study, thus restricting analysis to only a single timepoint. Choice of metabolite extraction [62,63] and tissue collection (i.e., form of euthanasia or anesthesia [64]) methods can impart systematic changes in the measured tissue metabolite levels compared to in vivo, especially for labile metabolites such as coenzymes [65], lactate, and succinate. Tissue collection steps from euthanasia to metabolism quenching with liquid nitrogen are time-critical and were limited to under 5 min per animal to limit effects on the metabolome [66] and to ensure changes were systematic in nature. Further, the animals were on dietary regimens for only six weeks, so the metabolic changes might not have occurred yet and therefore not show all significant changes associated with high-fat ad libitum and TRF diets. Some metabolites were not able to be consistently quantified across all tissues due to low biological abundance and poor signal on NMR spectra. However, major metabolites that showed significant differences across metabolic tissues due to dietary and sex effects were identified, hence demonstrating the potential use of multi-tissue NMR metabolomics for investigating efficient dietary regimens in combatting MetS. As there are currently no published metabolomics studies investigating multi-tissue and sex differences under TRF, we believe our study findings across multiple metabolic tissues can provide more insight into the metabolomic changes occurring due to TRF, and its effect on mitigating the development of MetS.

## 4. Materials and Methods

### 4.1. Animals

All animal experiments were approved by the Institutional Animal Care and Use Committee, and all animals were bred and housed individually in a USDA-approved facility at the University of Memphis. Male and female Nile grass rats (Arvicanthis niloticus) 12–18 months old were used for this study, with this age range corresponding to middle-age in humans—an age group greatly affected by MetS [1]. Throughout the study, animals were individually housed in Plexiglass cages with 20–30% humidity and temperature maintained at 22 ± 2 °C. Light exposure was set to 12 h light–dark cycle with lights on between 8:00 and 20:00.

Forty rats were divided into four dietary groups, with each group being age and sex matched (*n* = 10 per dietary group, male:female ratio 1:1). Prior to the study, all rats were fed global extruded rodent diet (chow, 2020X Teklad, 6.5% fat, 19.1% protein, and 47% carbohydrate) for two weeks for adaptation. At the beginning of the study, three animal groups were switched to an obesogenic diet that contained 60% fat (Research Diets, D12492, 35% fat (lard and soybean oil), 26% protein, and 26% carbohydrate (9.4% sucrose)). One group had access to the high-fat diet ad libitum (HF-AD), while two groups were only allowed access to the food for 6 h out of every 24 h. The early TRF group, HF-AM, had access to food for the first 6 h of the light cycle (8:00–14:00), and the late TRF group, HF-PM, had access to food during the second half of the light cycle (14:00–20:00). The fourth group remained on the global extruded rodent diet (chow) for the duration of the study. The chow group was used as a standard diet to compare the obesogenic effects of high-fat diet. All dietary groups had ad libitum access to water. Of note, during the study, there were seven random deaths that resulted in 3–5 animals per diet and sex. Six weeks after starting their respective protocols, the remaining animals were fasted overnight and euthanized by CO_2_ inhalation and cervical dislocation [67]. All animals were euthanized within a 2 h window. Heart, liver, and white adipose tissues were collected and immediately weighed, frozen in liquid nitrogen, and stored at −80 °C until processing for NMR analysis.

### 4.2. NMR Experiments

For heart and liver, ~50 and 100 mg, respectively, of frozen tissue was homogenized in 1.74 mL methanol using a Fisherbrand bead-mill homogenizer (Thermo Fisher Scientific, Waltham, MA, USA). Tissue homogenate was transferred to a centrifuge tube, and the lipid, aqueous, and protein components were separated using methyl tert-butyl ether (MTBE) liquid–liquid extraction [32,63]. The upper lipid (MTBE) layer and middle aqueous (MeOH/H_2_O) layer were extracted and transferred into glass vials. The remaining pellet was re-extracted to obtain any remnant lipid and aqueous portions by adding 1 mL of extraction solvent (MTBE:MeOH:H_2_O; 2.6/2.0/2.4–volume (*v*)/*v*/*v*). The lipid and aqueous fractions were separately dried under a stream of nitrogen. Lipids were resolubilized in 600 μL of a deuterated three solvent mixture of chloroform-d, methanol-d, and deuterium oxide (16:7:1–*v*/*v*/*v*) containing 1.18-mM dimethyl sulfone as an internal quantitative reference [32]. Aqueous metabolites were resolubilized in 600 μL of deuterium oxide buffered to a pH of 7.4 and containing 0.16-mM 3-(trimethylsilyl)propionic-2,2,3,3 acid as a quantitative reference [68]. Adipose tissue (~20 mg) was extracted following a slightly modified MTBE extraction that is specifically used for adipose samples [69].

Both lipid and aqueous spectra were obtained using a 400-MHz JEOL ECZ NMR spectrometer. Before acquiring spectra from lipid samples, the spectrometer was cooled to 0 °C to shift the water resonance to avoid overlap with most lipid peaks. For lipids, 16 FIDs (32 FIDs for liver) were recorded using a single-pulse proton sequence with water pre-saturation, a pulse angle of 45°, and a relaxation delay of 4 s. Aqueous metabolite measurements were acquired using a 1D-NOESY pulse sequence at ~22 °C with 16 FIDs, water pre-saturation, pulse angle of 90°, and relaxation delay of 15 s.

### 4.3. Multi-Tissue Metabolomics

Spectral processing for all samples was performed in JEOL Delta software (version 5.3.1, JEOL Ltd., Tokyo, Japan) using CRAFT [31]. Chemical shift fingerprints for lipids were determined using reference standards purchased from Nu-Chek Prep (St. Elysian, MN, USA), including trilinolein, tripalmitin, triolein, cholesterol, cholesteryl oleate, docosahexaenoic acid, 1-palmitoyl-2-hydroxy-sn-glycero-3-phosphocholine, 1,2-dipalmitoyl-sn-glycero3-phosphoethanolamine, 1,2-dioleoyl-sn-glycero-3-phosphocholine, and 1,2-dipalmitoyl-sn-glycero-3-phosphocholine, as well as sphingomyelin purchased from Matreya Inc. (State College, PA, USA). Chemical shift fingerprints for aqueous metabolites were determined by first using reference standards for L-lactic acid and L-leucine from MP Biomedicals (Solon, OH, USA), and alpha-D-glucose, creatinine, creatine monohydrate, L-valine, and L-alanine from Acros Organics (Geel, Belgium). Additional aqueous metabolites were identified using the human metabolome database (HMDB) [70] and relevant literature [62,65,71]. Chemical shifts for lipids and aqueous metabolites used for quantification are shown in Table 6. For all tissue samples, the same fingerprint for each metabolite was applied in CRAFT to systematically calculate the resonance amplitudes and quantify the metabolite. Metabolite concentrations were determined from the resonance amplitudes using the following formula:

(1) $$C_{x}=A_{x}\times \left(\frac{C_{\mathrm{ref}}}{A_{\mathrm{ref}}}\right)\times \left(\frac{N_{\mathrm{ref}}}{N_{x}}\right)$$

where C_x_ is analyte concentration, A_x_ is analyte peak area, N_x_ is the number of protons contributing to the analyte peak, and the subscript ‘ref’ denotes the corresponding quantities for the quantitative reference peak in each spectrum. In adipose tissue, aqueous metabolites were not profiled due to very low metabolite abundances.

Concentrations for SFA, UFA, and PUFA were calculated using a previously reported method of first estimating the molar percentage of UFA:

(2) $$\mathrm{UFA}\text{-}\%=\frac{\left[–\mathrm{CH}=\mathrm{CH}–\right]}{\left[–{\mathrm{CH}}_{3}\right]},$$

where the numerator corresponds to the amplitude of all olefinic acyl bonds (5.31–5.48 ppm), and the denominator is the amplitude of all terminal methyl groups (0.85–1.01 ppm) [72]. The NMR signals for both monounsaturated fatty acids (MUFA) at ~2.0 ppm and TFA at ~0.88 ppm directly overlap with cholesterol proton resonances, so the unambiguous total cholesterol peak at ~0.69 ppm was used to determine the contribution of the cholesterol protons in each spectrum, and the magnitude of overlap was subtracted from the MUFA and TFA amplitudes for calculating their correct concentrations. Some metabolite ratios such as SFA/UFA and Gmi/Gma were also calculated, as they are known to be potential biomarkers of metabolic disorder [73,74].

### 4.4. Statistical Analysis

Metabolite concentrations for all dietary and sex groups are presented as mean ± standard deviation (SD). Two-way analysis of variance (ANOVA) with Holm–Sidak test (Sigma Plot, version 14.5, SPSS Inc., Chicago, IL, USA) was used to compare metabolite concentrations in tissue samples between diets and sexes. Separate analyses were performed to independently examine the effects of the high-fat diet (HF-AD vs. chow) and TRF (HF-AM, HF-PM vs. HF-AD). In some aqueous samples, CRAFT was not able to detect some metabolite peaks due to low signal-to-noise ratio, and these missing metabolite amplitudes were replaced using an estimated detection limit of 1/5 of the minimum positive amplitude measured for the respective metabolite in all aqueous samples from a given tissue. Heatmaps were created for each tissue, showing differences in metabolite levels between groups using auto-scaled data (mean-subtracted and divided by standard deviation) in MetaboAnalyst (https://www.metaboanalyst.ca/, accessed on 7 January 2022). Correlation between metabolite level and metadata (sex or dietary group) was assessed by partial correlation analysis in MetaboAnalyst by analyzing for metabolite correlations with sex controlled for dietary group and vice versa. Independent partial correlation analysis was performed for comparing HF-AD to each TRF dietary group. Spearman rank was used as the correlation measure for all partial correlation analyses. For all statistical tests, a *p*-value < 0.05 was considered as showing statistical difference. Of note, no multiple-testing correction was performed to adjust *p*-values for two-way ANOVA or partial correlation analysis, as the metabolic variables considered in our study are scientifically sensible comparisons rather than randomly selected variables [75,76], and further multiple-testing correction might cause notable effects to be insignificant (i.e., accentuates type II error [76,77,78]) due to limited sample size in this study [79].

## 5. Conclusions

Time-domain NMR metabolomics revealed lipid remodeling in liver, heart, and adipose tissues, and ectopic accumulation of cardiac and hepatic lipids for ad libitum high-fat feeding compared to a standard chow diet. Many of these metabolic effects of high-fat diet were attenuated by TRF, with animals eating a high-fat TRF diet expressing lower lipid levels in the heart and liver than their ad libitum-fed counterparts. Regardless of diet, females harbored greater amounts of hepatic lipids compared to males. Hence, this study demonstrates the potential of multi-tissue, robust, time-domain NMR metabolomics in understanding the etiology of MetS and TRF.

## Figures and Tables

**Table 1:** Hepatic, cardiac, and adipose lipid concentrations (mM) indicated as mean ± standard deviation for both male and female chow and high-fat ad libitum (HF-AD) groups.

	Liver						Heart						Adipose					
	Male		Female		*p*-Values		Male		Female		*p*-Values		Male		Female		*p*-Values	
	Chow (*n* =3 )	HF-AD (*n* = 5)	Chow (*n* = 3)	HF-AD (*n* = 5)	Sex	Diet	Chow (*n* = 4)	HF-AD (*n* = 5)	Chow (*n* = 3)	HF-AD (*n* = 5)	Sex	Diet	Chow (*n* = 4)	HF-AD (*n* = 5)	Chow (*n* = 3)	HF-AD (*n* = 5)	Sex	Diet
Om3	4.4 ± 1.2	5.7 ± 2	9.5 ± 1	8.2 ± 2.3	**0.002 ^f^**	1.00	0.91 ± 0.16	0.77 ± 0.17	0.87 ± 0.04	0.88 ± 0.2	0.54	0.36	1.91 ± 0.96	1.05 ± 0.44	1.34 ± 0.29	0.88 ± 0.1	0.19	**0.028 ⁰**
Tg	7.9 ± 3.1	30.6 ± 15.8	30.8 ± 10.1	68.6 ± 21.8	**0.003 ^f^**	0.003 *	0.12 ± 0.12	0.36 ± 0.12	0.2 ± 0.06	0.35 ± 0.2	0.68	0.017 *	22.2 ± 11.6	22.8 ± 2.9	20.9 ± 3.6	21.8 ± 3.7	0.72	0.81
TFA	49.6 ± 8	103.3 ± 37.5	121.4 ± 21.6	205.8 ± 41.7	**<0.001 ^f^**	0.002 *	8.2 ± 0.7	10.2 ± 1.3	8.3 ± 0.12	10.5 ± 0.61	0.51	<0.001 *	66.2 ± 32.4	68.2 ± 11.6	63.9 ± 12.1	67.7 ± 13.3	0.89	0.76
LA	6.1 ± 0.8	14.3 ± 5.7	18.6 ± 7.4	51.5 ± 39.5	0.06	0.11	0.39 ± 0.08	0.32 ± 0.06	0.31 ± 0.09	0.42 ± 0.07	0.88	0.26	33.8 ± 15.6	25.4 ± 3.4	30.9 ± 6.1	24.4 ± 3.7	0.10	0.65
UFA	32.4 ± 4.8	63.7 ± 20.1	86.6 ± 21.8	130.1 ± 25.4	**0.005 ^f^**	<0.001 *	6.2 ± 0.7	6.55 ± 1	5.4 ± 0.89	7.3 ± 0.98	0.68	0.06	51.2 ± 24.1	47.8 ± 14.2	44.7 ± 7.5	43.9 ± 10.6	0.51	0.79
SFA	17.1 ± 3.7	39.6 ± 18.5	34.8 ± 1.8	75.7 ± 20.6	**0.007 ^f^**	0.002 *	2.0 ± 0.3	3.6 ± 0.66	2.8 ± 0.77	3.1 ± 0.78	0.77	0.007 *	15.0 ± 11.3	20.3 ± 4.2	19.2 ± 6.3	23.8 ± 3.7	0.27	0.16
MUFA	7.6 ± 2.5	28.4 ± 14.4	26.1 ± 5.4	63.8 ± 12.7	**<0.001 ^f^**	<0.001 *	0.90 ± 0.2	1.4 ± 0.22	0.7 ± 0.01	1.3 ± 0.22	0.30	<0.001 *	16.4 ± 6.6	24 ± 1.8	20 ± 4.4	25 ± 4.7	0.32	0.016 *
PUFA	24.8 ± 3.7	35.3 ± 9	60.5 ± 17.1	66.3 ± 14.5	**<0.001 ^f^**	0.22	5.3 ± 0.7	5.2 ± 0.9	4.7 ± 0.88	6.0 ± 0.88	0.49	0.35	34.9 ± 17.7	23.9 ± 14.2	24.7 ± 5.7	18.9 ± 6	0.23	0.19
UFA-%	66 ± 3%	63 ± 7%	71 ± 6%	63 ± 5%	0.36	0.11	75 ± 4%	64 ± 5%	66 ± 10%	70 ± 8%	0.80	0.18	77 ± 10%	69 ± 8%	70 ± 5%	64 ± 4%	0.13	0.08
SFA-%	34 ± 3%	37 ± 7%	29 ± 6%	37 ± 5%	0.36	0.11	25 ± 4%	36 ± 5%	34 ± 10%	30 ± 8%	0.80	0.18	18 ± 10%	31 ± 8%	30 ± 5%	36 ± 4%	0.13	0.08
MUFA-%	15 ± 3%	26 ± 7%	21 ± 1%	31 ± 1%	**0.013 ^f^**	<0.001 *	11 ± 2%	14 ± 2%	9 ± 0.1%	12 ± 2%	0.11	0.005 *	21 ± 9%	36 ± 5%	31 ± 2%	37 ± 1%	**0.040 ^f^**	0.001 *
PUFA-%	50 ± 6%	37 ± 6%	49 ± 7%	32 ± 5%	0.54	**0.004 ⁰**	64 ± 6%	51 ± 5%	57 ± 10%	57 ± 7%	0.80	**0.033 ⁰**	44 ± 21%	33 ± 13%	39 ± 8%	27 ± 4%	0.40	0.11
SFA/UFA	0.53 ± 0.08	0.6 ± 0.16	0.42 ± 0.16	0.59 ± 0.13	0.40	0.11	0.33 ± 0.08	0.56 ± 0.13	0.54 ± 0.23	0.44 ± 0.18	0.81	0.22	0.31 ± 0.15	0.46 ± 0.15	0.43 ± 0.11	0.56 ± 0.10	0.13	0.06
TC	1.9 ± 0.3	5.1 ± 1.5	2.8 ± 1	4.3 ± 2.9	0.95	0.035 *	0.39 ± 0.04	0.40 ± 0.06	0.36 ± 0.02	0.42 ± 0.04	0.66	0.15	-	-	-	-	-	-
PC	3.2 ± 0.3	2.8 ± 0.5	3.4 ± 0.4	2.5 ± 0.7	0.89	**0.031 ⁰**	1.31 ± 0.14	1.41 ± 0.14	1.13 ± 0.02	1.46 ± 0.15	0.62	0.022 *	-	-	-	-	-	-
PE	1.8 ± 0.2	1.7 ± 0.3	1.6 ± 0.3	1.4 ± 0.8	0.40	0.64	1.29 ± 0.17	1.16 ± 0.77	1.08 ± 0.06	1.38 ± 0.32	0.69	0.43	-	-	-	-	-	-
SM	0.3 ± 0	0.3 ± 0.1	0.2 ± 0.1	0.2 ± 0.1	0.24	0.45	0.06 ± 0.01	0.08 ± 0.04	0.06 ± 0.01	0.08 ± 0.01	0.72	0.18	-	-	-	-	-	-
DHA	-	-	-	-	-	-	1.1 ± 0.3	1.1 ± 0.06	1.1 ± 0.06	1.3 ± 0.25	0.52	0.93	**-**	-	-	-	-	-

Two-way ANOVA was used to determine main effects of sex, diet, and interaction on lipid compositions with Holm–Sidak post-hoc testing. Bolded cells show significance using the following symbols to denote differences: * for HF-AD higher than chow, **⁰** for HF-AD lower than chow, **^f^** for female higher than males. Abbreviations: Tg = triglycerides; PC = phosphatidylcholine; SM = sphingomyelin; PE = phosphatidylethanolamine; LA = linoleic acid; DHA = docosahexaenoic acid; MUFA = monounsaturated fatty acids; PUFA = polyunsaturated fatty acids; UFA = unsaturated fatty acids; SFA = saturated fatty acids; Om3 = omega-3 fatty acids; TFA = total fatty acids; TC = total cholesterol.

**Table 2:** Hepatic and cardiac aqueous metabolite concentrations (mM) indicated as mean ± standard deviation for both male and female chow and high-fat ad libitum (HF-AD) groups.

	Liver						Heart					
	Male		Female		*p*-Values		Male		Female		*p*-Values	
	Chow (*n* = 3)	HF-AD (*n* = 5)	Chow (*n* = 3)	HF-AD (*n* = 5)	Sex	Diet	Chow (*n* = 4)	HF-AD (*n* = 5)	Chow (*n* = 3)	HF-AD (*n* = 5)	Sex	Diet
Glucose	12.5 ± 1.2	9.9 ± 3.1	5.1 ± 1.8	5.9 ± 2.3	**<0.001 ^m^**	0.47	0.28 ± 0.06	0.43 ± 0.23	0.21 ± 0.02	3.81 ± 7.65	0.45	0.40
Lactate	2.2 ± 0.3	1.3 ± 0.2	1.5 ± 0.4	1.8 ± 0.9	0.71	0.37	1.40 ± 0.20	1.27 ± 0.40	1.42 ± 0.36	2.61 ± 2.22	0.36	0.43
Alanine	0.58 ± 0.12	0.43 ± 0.16	0.48 ± 0.07	0.42 ± 0.08	0.36	0.10	0.21 ± 0.05	0.20 ± 0.10	0.19 ± 0.05	0.35 ± 0.27	0.53	0.48
Acetate	0.27 ± 0.03	0.19 ± 0.02	0.2 ± 0.04	0.15 ± 0.04	**0.006 ^m^**	**0.002 ⁰**	0.02 ± 0.03	0.02 ± 0.003	0.02 ± 0.01	0.04 ± 0.04	0.48	0.33
Choline	0.10 ± 0.08	0.12 ± 0.06	0.13 ± 0.05	0.10 ± 0.05	0.80	0.75	0.01 ± 0.08	0.01 ± 0.004	0.01 ± 0.002	0.03 ± 0.03	0.35	0.37
Creatine	0.31 ± 0.27	0.20 ± 0.13	0.18 ± 0.07	0.07 ± 0.03	0.09	0.16	0.77 ± 0.17	0.62 ± 0.08	0.78 ± 0.18	0.67 ± 0.34	0.81	0.28
Inosine	1.01 ± 0.16	0.72 ± 0.25	0.99 ± 0.18	0.51 ± 0.16	0.28	**0.003 ⁰**	0.32 ± 0.09	0.27 ± 0.07	0.33 ± 0.09	0.32 ± 0.13	0.54	0.52
Fumarate	0.07 ± 0.02	0.07 ± 0.04	0.04 ± 0.002	0.04 ± 0.02	**0.042 ^m^**	0.80	0.03 ± 0.01	0.02 ± 0.01	0.02 ± 0.002	0.03 ± 0.005	0.63	0.22
Succinate	0.71 ± 0.16	0.63 ± 0.21	0.5 ± 0.11	0.26 ± 0.22	**0.015 ^m^**	0.13	0.08 ± 0.01	0.07 ± 0.03	0.08 ± 0.05	0.07 ± 0.01	0.93	0.24
Glutamine	1.9 ± 0.3	1.5 ± 0.3	1.3 ± 0.1	0.7 ± 0.3	**<0.001 ^m^**	**0.003 ⁰**	0.32 ± 0.09	0.35 ± 0.11	0.38 ± 0.15	0.34 ± 0.05	0.54	0.95
Glutamate	1.2 ± 0.3	1.1 ± 0.4	1.1 ± 0.3	0.8 ± 0.5	0.53	0.35	0.45 ± 0.05	0.35 ± 0.06	0.44 ± 0.09	0.38 ± 0.15	0.82	0.11
Gmi/Gma	1.7 ± 0.2	1.5 ± 0.6	1.2 ± 0.2	1.3 ± 1.0	0.37	0.93	0.71 ± 0.23	1.03 ± 0.31	0.86 ± 0.23	1.0 ± 0.33	0.69	0.13
Aspartate	0.08 ± 0.01	0.06 ± 0.01	0.04 ± 0.03	0.06 ± 0.03	0.15	0.86	0.13 ± 0.07	0.15 ± 0.01	0.25 ± 0.10	0.25 ± 0.06	**0.003 ^f^**	0.68
3-hydroxybutyrate	0.71 ± 0.02	0.12 ± 0.05	0.13 ± 0.03	0.14 ± 0.21	0.73	0.84	0.03 ± 0.01	0.04 ± 0.02	0.02 ± 0.01	0.05 ± 0.05	0.81	0.13
Valine	0.14 ± 0.02	0.11 ± 0.02	0.14 ± 0.07	0.09 ± 0.02	0.71	**0.024 ⁰**	-	-	-	-	-	-
Isoleucine	0.11 ± 0.03	0.11 ± 0.12	0.35 ± 0.07	0.04 ± 0.01	0.05	**0.002 ⁰**	-	-	-	-	-	-
Formate	0.04 ± 0.04	0.08 ± 0.06	0.05 ± 0.01	0.05 ± 0.03	0.50	0.39	-	-	-	-	-	-
Histidine	0.11 ± 0.03	0.09 ± 0.02	0.08 ± 0.04	0.06 ± 0.01	**0.025 ^m^**	0.14	-	-	-	-	-	-
Creatinine	0.03 ± 0.03	0.04 ± 0.01	0.06 ± 0.01	0.02 ± 0.02	0.49	0.06	-	-	-	-	-	-
Leucine	0.17 ± 0.07	0.11 ± 0.05	0.14 ± 0.05	0.09 ± 0.04	0.38	**0.047 ⁰**	-	-	-	-	-	-
Niacinamide	0.10 ± 0.04	0.07 ± 0.01	0.07 ± 0.01	0.05 ± 0.02	**0.038 ^m^**	**0.025 ⁰**	-	-	-	-	-	-
Phenylalanine	0.05 ± 0.01	0.03 ± 0.02	0.03 ± 0.01	0.02 ± 0.02	**0.033 ^m^**	0.07	-	-	-	-	-	-
Tyrosine	0.04 ± 0.01	0.03 ± 0.01	0.03 ± 0.003	0.01 ± 0.01	**0.010 ^m^**	**0.013 ⁰**	-	-	-	-	-	-
Hypoxanthine	-	-	-	-	-	-	0.04 ± 0.01	0.05 ± 0.01	0.05 ± 0.01	0.09 ± 0.08	0.35	0.27
Methionine	-	-	-	-	-	-	0.05 ± 0.02	0.04 ± 0.02	0.04 ± 0.02	0.06 ± 0.03	0.66	0.93
Taurine	-	-	-	-	-	-	0.74 ± 0.28	0.63 ± 0.21	0.63 ± 0.21	0.78 ± 0.21	0.67	0.94

Two-way ANOVA was used to determine main effects of sex, diet, and interaction on aqueous compositions with Holm-Sidak post-hoc testing. Bolded cells show significance using the following symbols to denote differences: **⁰** for HF-AD lower than chow, **^m^** for male higher than females, **^f^** for female higher than males. Abbreviations: Gmi/Gma = ratio of glutamine to glutamate.

**Table 3:** Hepatic metabolite concentrations (mM) indicated as mean ± standard deviation for both male and female high-fat ad libitum (HF-AD), high-fat morning time-restricted (HF-AM), and high-fat evening time-restricted (HF-PM) groups.

	Male			Females			*p*-Values	
	HF-AD (*n* = 5)	HF-AM (*n* = 4)	HF-PM (*n* = 4)	HF-AD (*n* = 5)	HF-AM (*n* = 4)	HF-PM (*n* = 4)	Sex	Diet
Omega-3	5.66 ± 2.03	4.79 ± 2.01	4.15 ± 1.89	8.21 ± 2.28	5.48 ± 2.45	6.10 ± 1.71	**0.049 ^f^**	0.13
Triglycerides	30.6 ± 15.8	17.9 ± 5.9	17.7 ± 9.9	68.6 ± 21.8	27.9 ± 18.5	45.56 ± 20.8	**0.001 ^f^**	0.009 *
TFA	103.3 ± 37.5	90.0 ± 19.9	62.8 ± 28.1	205.8 ± 41.7	107.1 ± 51.1	148.4 ± 54.1	**<0.001 ^f^**	0.015 ⁰^,^*
Linoleic Acid	14.3 ± 5.7	10.3 ± 6.6	15.5 ± 12.1	51.4 ± 39.5	22.0 ± 11.3	38.5 ± 30.4	**0.014 ^f^**	0.31
UFA	63.7 ± 20.1	55.5 ± 13.6	38.9 ± 13.9	130.0 ± 25.4	73.4 ± 34.2	95.3 ± 35.2	**<0.001 ^f^**	0.020 ⁰^,^*
SFA	39.6 ± 18.5	34.4 ± 7.1	23.9 ± 14.3	75.7 ± 20.6	33.6 ± 16.9	53.1 ± 19.1	**0.004 ^f^**	0.017 *
MUFA	28.4 ± 14.4	19.6 ± 6.2	20.6 ± 10.2	63.7 ± 12.7	31.6 ± 16.7	45.6 ± 18.3	**<0.001 ^f^**	0.015 *
PUFA	35.3 ± 9.0	35.9 ± 11.1	18.2 ± 10.1	66.2 ± 14.5	41.8 ± 17.6	49.7 ± 17.0	**<0.001 ^f^**	**0.041 ⁰**
UFA-%	62 ± 7%	62 ± 3%	64 ± 6%	63 ± 6%	69 ± 2%	63 ± 2%	0.18	0.64
SFA-%	37 ± 7%	38 ± 3%	35 ± 6%	36 ± 5%	31 ± 2%	36 ± 2%	0.18	0.64
MUFA-%	26 ± 6%	22 ± 6%	34 ± 17%	31 ± 1%	28 ± 3%	30 ± 3%	0.48	0.20
PUFA-%	36 ± 12%	39 ± 4%	29 ± 17%	32 ± 5%	40 ± 4%	33 ± 2%	0.91	0.20
SFA/UFA	0.60 ± 0.16	0.62 ± 0.07	0.57 ± 0.16	0.58 ± 0.13	0.45 ± 0.03	0.56 ± 0.05	0.16	0.60
Total Cholesterol	5.11 ± 1.47	3.55 ± 1.03	3.52 ± 1.47	4.32 ± 2.88	2.11 ± 0.04	2.79 ± 0.89	0.14	0.05
Phosphatidylcholine	2.80 ± 0.49	3.25 ± 0.33	2.76 ± 0.15	2.48 ± 0.67	2.98 ± 0.45	2.62 ± 0.81	0.26	0.16
Phosphatidylethanolamine	1.69 ± 0.32	1.49 ± 0.16	1.33 ± 0.67	1.42 ± 0.83	1.46 ± 0.12	1.37 ± 0.63	0.69	0.73
Sphingomyelin	0.25 ± 0.11	0.37 ± 0.13	0.25 ± 0.09	0.19 ± 0.08	0.21 ± 0.05	0.18 ± 0.07	**0.018 ^m^**	0.20
Glucose	9.13 ± 2.85	11.01 ± 3.61	10.1 ± 2.56	5.49 ± 2.17	8.58 ± 3.58	11.3 ± 3.89	0.20	0.08
Lactate	1.34 ± 0.23	1.54 ± 0.65	1.47 ± 0.46	1.77 ± 0.95	1.24 ± 0.40	2.51 ± 1.07	0.17	0.22
Alanine	0.42 ± 0.16	0.56 ± 0.08	0.60 ± 0.15	0.41 ± 0.08	0.48 ± 0.12	0.65 ± 0.27	0.81	**0.032 ^‡^**
Acetate	0.19 ± 0.02	0.25 ± 0.05	0.21 ± 0.05	0.15 ± 0.04	0.24 ± 0.07	0.26 ± 0.07	0.92	**0.006 ^+,‡^**
Choline	0.11 ± 0.06	0.11 ± 0.03	0.14 ± 0.03	0.10 ± 0.05	0.12 ± 0.05	0.10 ± 0.03	0.80	0.67
Creatine	0.20 ± 0.13	0.10 ± 0.04	0.15 ± 0.15	0.073 ± 0.03	0.08 ± 0.02	0.07 ± 0.03	**0.031 ^m^**	0.49
Inosine	0.24 ± 0.08	0.26 ± 0.09	0.30 ± 0.06	0.17 ± 0.05	0.26 ± 0.10	0.27 ± 0.06	0.26	0.06
Fumarate	0.07 ± 0.04	0.06 ± 0.02	0.11 ± 0.03	0.043 ± 0.02	0.06 ± 0.02	0.10 ± 0.04	0.38	**0.003 ^‡‡^**
Succinate	0.62 ± 0.21	0.44 ± 0.06	0.66 ± 0.09	0.26 ± 0.22	0.64 ± 0.27	0.57 ± 0.022	0.28	0.19
Glutamine	1.45 ± 0.32	1.28 ± 0.23	0.99 ± 0.41	0.74 ± 0.25	0.91 ± 0.39	0.95 ± 0.19	**0.006 ^m^**	0.64
Glutamate	1.06 ± 0.36	1.23 ± 0.54	1.61 ± 0.67	0.80 ± 0.54	1.67 ± 0.91	1.30 ± 0.84	0.95	0.15
Gmi/Gma	1.49 ± 0.56	1.25 ± 0.70	0.63 ± 0.13	1.33 ± 0.30	0.89 ± 0.90	0.91 ± 0.52	0.78	0.19
Aspartate	0.058 ± 0.01	0.08 ± 0.03	0.08 ± 0.02	0.06 ± 0.03	0.06 ± 0.03	0.06 ± 0.03	0.29	0.58
3-hydroxybutyrate	0.12 ± 0.05	0.14 ± 0.04	0.05 ± 0.01	0.13 ± 0.21	0.05 ± 0.02	0.05 ± 0.02	0.55	0.30
Valine	0.10 ± 0.02	0.12 ± 0.04	0.15 ± 0.06	0.09 ± 0.02	0.15 ± 0.12	0.23 ± 0.23	0.48	0.16
Isoleucine	0.11 ± 0.12	0.08 ± 0.04	0.07 ± 0.05	0.04 ± 0.01	0.10 ± 0.13	0.21 ± 0.13	0.63	0.66
Formate	0.08 ± 0.06	0.06 ± 0.03	0.06 ± 0.02	0.05 ± 0.03	0.09 ± 0.06	0.11 ± 0.09	0.51	0.69
Histidine	0.04 ± 0.01	0.05 ± 0.02	0.06 ± 0.03	0.03 ± 0.01	0.05 ± 0.01	0.05 ± 0.01	0.12	0.09
Creatinine	0.04 ± 0.01	0.03 ± 0.02	0.04 ± 0.03	0.03 ± 0.02	0.03 ± 0.01	0.03 ± 0.01	0.12	0.46
Leucine	0.10 ± 0.05	0.15 ± 0.08	0.12 ± 0.05	0.09 ± 0.04	0.09 ± 0.04	0.13 ± 0.07	0.23	0.46
Niacinamide	0.02 ± 0.003	0.03 ± 0.01	0.02 ± 0.005	0.02 ± 0.01	0.02 ± 0.001	0.02 ± 0.01	**0.028 ^m^**	0.05
Phenylalanine	0.03 ± 0.02	0.06 ± 0.02	0.04 ± 0.02	0.02 ± 0.01	0.03 ± 0.001	0.04 ± 0.02	**0.022 ^m^**	0.10
Tyrosine	0.027 ± 0.01	0.05 ± 0.01	0.04 ± 0.01	0.02 ± 0.01	0.02 ± 0.01	0.03 ± 0.002	**<0.001 ^m^**	**0.034 ^+^**

Two-way ANOVA was used to determine main effects of sex, diet, and interaction on metabolite compositions with Holm–Sidak post-hoc testing. Bolded cells show significance using the following symbols to denote differences: * for HF-AM lower than HF-AD, ⁰ for HF-PM lower than HF-AD, ^+^ for HF-AM higher than HF-AD, ^‡^ for HF-PM higher than HF-AD, ^‡‡^ for HF-PM higher than HF-AD and HF-AM, **^f^** for female higher than males, **^m^** for male higher than females. Abbreviations: Omega-3 = omega-3 fatty acids; MUFA = monounsaturated fatty acids; PUFA = polyunsaturated fatty acids; UFA = unsaturated fatty acids; SFA = saturated fatty acids; TFA = total fatty acids; Gmi/Gma = ratio of glutamine to glutamate.

**Table 4:** Cardiac metabolite concentrations (mM) indicated as mean ± standard deviation for both male and female high-fat ad libitum (HF-AD), high-fat morning time-restricted (HF-AM), and high-fat evening time-restricted (HF-PM) groups.

	Male			Female			*p*-Values	
	HF-AD (*n* = 5)	HF-AM (*n* = 4)	HF-PM (*n* = 4)	HF-AD (*n* = 5)	HF-AM (*n* = 4)	HF-PM (*n* = 4)	Sex	Diet
Omega-3	0.77 ± 0.17	0.69 ± 0.17	0.73 ± 0.04	0.88 ± 0.2	0.82 ± 0.24	0.71 ± 0.09	0.27	0.40
Triglycerides	0.36 ± 0.12	0.2 ± 0.12	0.18 ± 0.12	0.35 ± 0.2	0.29 ± 0.26	0.28 ± 0.18	0.38	0.26
TFA	10.16 ± 1.26	8.89 ± 0.44	8.17 ± 0.75	10.46 ± 0.61	8.63 ± 1.44	8.68 ± 0.62	0.63	<0.001 ⁰^,^*
Linoleic Acid	1.38 ± 0.28	1.3 ± 0.26	1.04 ± 0.1	1.25 ± 0.25	0.89 ± 0.08	1.3 ± 0.15	0.27	0.10
UFA	6.55 ± 1	6.15 ± 0.89	5.62 ± 0.49	7.33 ± 0.98	5.99 ± 1.21	6.1 ± 0.71	0.32	**0.046**
SFA	3.62 ± 0.66	2.74 ± 0.56	2.55 ± 0.81	3.13 ± 0.78	2.64 ± 0.48	2.58 ± 0.51	0.48	**0.033 ⁰**
MUFA	1.37 ± 0.22	1.22 ± 0.11	0.98 ± 0.2	1.33 ± 0.22	0.98 ± 0.24	1.15 ± 0.27	0.66	0.018 ⁰^,^*
PUFA	5.17 ± 0.9	4.93 ± 0.95	4.64 ± 0.56	6 ± 0.88	5.02 ± 1.0	4.95 ± 0.53	0.23	0.13
UFA-%	64 ± 5%	69 ± 7%	69 ± 8%	70 ± 8%	69 ± 5%	70 ± 6%	0.38	0.71
SFA-%	36 ± 5%	31 ± 7%	31 ± 8%	30 ± 8%	31 ± 5%	30 ± 6%	0.38	0.71
MUFA-%	14 ± 2%	14 ± 2%	12 ± 2%	13 ± 2%	11 ± 1%	13 ± 3%	0.37	0.73
PUFA-%	51 ± 5%	55 ± 8%	57 ± 10%	57 ± 7%	58 ± 4%	57 ± 4%	0.27	0.58
SFA/UFA	0.56 ± 0.13	0.46 ± 0.15	0.46 ± 0.17	0.44 ± 0.18	0.45 ± 0.09	0.43 ± 0.12	0.37	0.68
Total Cholesterol	0.4 ± 0.06	0.37 ± 0.07	0.36 ± 0.03	0.42 ± 0.04	0.38 ± 0.03	0.39 ± 0.04	0.39	0.24
Phosphatidylcholine	1.41 ± 0.14	1.3 ± 0.18	1.22 ± 0.08	1.46 ± 0.15	1.28 ± 0.15	1.25 ± 0.17	0.74	**0.033 ⁰**
Phosphatidylethanolamine	1.16 ± 0.77	0.81 ± 0.39	0.75 ± 0.45	1.38 ± 0.32	0.82 ± 0.11	1.34 ± 0.68	0.20	0.19
Sphingomyelin	0.08 ± 0.04	0.09 ± 0.02	0.06 ± 0.01	0.08 ± 0.01	0.07 ± 0.01	0.08 ± 0.01	0.31	0.35
Glucose	0.43 ± 0.23	0.22 ± 0.13	0.33 ± 0.1	3.81 ± 7.65	0.32 ± 0.32	0.23 ± 0.2	0.39	0.40
Lactate	1.27 ± 0.4	1.36 ± 0.66	1.43 ± 0.55	2.61 ± 2.22	1.59 ± 0.52	1.63 ± 0.49	0.20	0.51
Alanine	0.2 ± 0.1	0.23 ± 0.14	0.25 ± 0.1	0.35 ± 0.27	0.24 ± 0.06	0.23 ± 0.08	0.45	0.75
Acetate	0.02 ± 0.003	0.02 ± 0.01	0.03 ± 0.01	0.04 ± 0.04	0.02 ± 0.002	0.03 ± 0.01	0.46	0.48
Choline	0.01 ± 0.004	0.01 ± 0.01	0.01 ± 0.003	0.03 ± 0.03	0.02 ± 0.003	0.01 ± 0.004	0.12	0.43
Creatine	0.62 ± 0.08	0.73 ± 0.31	0.81 ± 0.17	0.67 ± 0.34	0.77 ± 0.14	0.85 ± 0.14	0.75	0.30
Inosine	0.27 ± 0.07	0.28 ± 0.09	0.39 ± 0.1	0.32 ± 0.13	0.36 ± 0.06	0.37 ± 0.08	0.39	0.21
Fumarate	0.02 ± 0.01	0.02 ± 0.01	0.02 ± 0.005	0.03 ± 0.005	0.02 ± 0.003	0.03 ± 0.01	0.16	0.49
Succinate	0.07 ± 0.03	0.11 ± 0.06	0.06 ± 0.01	0.07 ± 0.01	0.09 ± 0.03	0.07 ± 0.03	0.61	0.06
Glutamine	0.35 ± 0.11	0.34 ± 0.08	0.33 ± 0.09	0.34 ± 0.05	0.33 ± 0.17	0.36 ± 0.16	1.00	0.97
Glutamate	0.35 ± 0.06	0.36 ± 0.12	0.48 ± 0.14	0.38 ± 0.15	0.44 ± 0.12	0.58 ± 0.16	0.28	0.08
Gmi/Gma	1.03 ± 0.31	0.97 ± 0.21	0.73 ± 0.17	1.0 ± 0.33	0.8 ± 0.49	0.59 ± 0.17	0.45	0.09
Aspartate	0.15 ± 0.01	0.17 ± 0.04	0.4 ± 0.16	0.25 ± 0.06	0.29 ± 0.07	0.4 ± 0.05	**0.034 ^f^**	**<0.001 ^‡‡^**
3-hydroxybutyrate	0.04 ± 0.02	0.03 ± 0.01	0.02 ± 0.01	0.05 ± 0.05	0.15 ± 0.26	0.04 ± 0.01	0.23	0.44
Hypoxanthine	0.05 ± 0.02	0.04 ± 0.02	0.07 ± 0.02	0.09 ± 0.08	0.05 ± 0.02	0.05 ± 0.03	0.55	0.46
Methionine	0.04 ± 0.01	0.04 ± 0.02	0.05 ± 0.02	0.06 ± 0.03	0.05 ± 0.03	0.09 ± 0.05	0.23	0.50
Taurine	0.57 ± 0.15	0.56 ± 0.21	0.72 ± 0.17	0.78 ± 0.21	0.71 ± 0.12	0.68 ± 0.14	0.20	0.86
DHA	0.32 ± 0.06	0.29 ± 0.08	0.28 ± 0.07	0.42 ± 0.07	0.31 ± 0.1	0.3 ± 0.06	0.11	0.05

Two-way ANOVA was used to determine main effects of sex, diet, and interaction on metabolite compositions with Holm–Sidak post-hoc testing. Bolded cells show significance using the following symbols to denote differences: * for HF-AM lower than HF-AD, ⁰ for HF-PM lower than HF-AD, ^‡‡^ for HF-PM higher than HF-AD and HF-AM, **^f^** for female higher than males. Interaction was determined for cardiac linoleic acid, which was lower in HF-AM females compared to both HF-AD females and HF-AM males. Abbreviations: Omega-3 = omega-3 fatty acids, DHA = docosahexaenoic acid; MUFA = monounsaturated fatty acids; PUFA = polyunsaturated fatty acids; UFA = unsaturated fatty acids; SFA, saturated fatty acids; TFA = total fatty acids; Gmi/Gma = ratio of glutamine to glutamate.

**Table 5:** Adipose tissue metabolite concentrations (mM) indicated as mean ± standard deviation for both male and female high-fat ad libitum (HF-AD), high-fat morning time-restricted (HF-AM), and high-fat evening time-restricted (HF-PM) groups.

	Male			Female			*p*-Values	
	HF-AD (*n* = 5)	HF-AM (*n* = 4)	HF-PM (*n* =4)	HF-AD (*n* = 5)	HF-AM (*n* = 3)	HF-PM (*n* = 4)	Sex	Diet
Omega-3	1.05 ± 0.44	1.08 ± 0.18	0.94 ± 0.24	0.88 ± 0.1	0.99 ± 0.06	0.84 ± 0.22	0.26	0.55
Triglycerides	22.8 ± 2.9	23.7 ± 1.1	22.4 ± 5	21.8 ± 3.7	25.8 ± 3.1	17.9 ± 4.4	0.46	0.08
TFA	68.2 ± 11.6	68.4 ± 3.6	61.1 ± 11.3	67.7 ± 13.3	72.6 ± 10.2	57.8 ± 14.6	0.98	0.17
Linoleic Acid	25.4 ± 3.4	23 ± 4.5	21 ± 6.1	24.4 ± 3.7	27.2 ± 4.6	19.7 ± 8.5	0.75	0.15
UFA	47.8 ± 14.2	45 ± 7.1	39.3 ± 9.9	43.9 ± 10.6	46.8 ± 6.1	37.7 ± 9	0.78	0.27
SFA	20.3 ± 4.2	23.4 ± 6.8	21.8 ± 2.4	23.8 ± 3.7	25.8 ± 4.1	20.1 ± 5.7	0.48	0.33
MUFA	24 ± 1.8	26.2 ± 1.4	23.5 ± 4.6	25 ± 4.7	24 ± 1.9	20.2 ± 5.5	0.34	0.21
PUFA	23.9 ± 14.2	18.7 ± 8.4	15.8 ± 5.6	18.9 ± 6	22.9 ± 5.9	17.5 ± 3.9	0.93	0.48
UFA-%	69 ± 8%	66 ± 10%	64 ± 5%	64 ± 4%	65 ± 1%	66 ± 1%	0.58	0.72
SFA-%	30 ± 8%	34 ± 10%	36 ± 5%	36 ± 4%	35 ± 1%	34 ± 1%	0.58	0.72
MUFA-%	35 ± 5%	38 ± 3%	38 ± 2%	37 ± 1%	33 ± 4%	34 ± 3%	0.07	0.91
PUFA-%	33 ± 13%	27 ± 12%	25 ± 5%	27 ± 4%	31 ± 4%	31 ± 3%	0.82	0.75
SFA/UFA	0.46 ± 0.15	0.54 ± 0.2	0.58 ± 0.11	0.56 ± 0.1	0.55 ± 0.03	0.53 ± 0.03	0.72	0.73

No statistically significant differences in metabolite concentrations in response to TRF were demonstrated in adipose samples using two-way ANOVA. Abbreviations: Omega-3 = omega-3 fatty acids, TFA = total fatty acids, PUFA = polyunsaturated fatty acids, MUFA = monounsaturated fatty acids, UFA = unsaturated fatty acids, SFA = saturated fatty acids.

**Table 6:** ^1^H-NMR chemical shift assignment for all quantified metabolites. For organ specification: L = liver, H = heart, and A = adipose tissue.

Metabolite	Chemical Shift (ppm) and Multiplicity	Protons (*n*)	Organs
3-hydroxybutyrate	1.07 (d)	3	L, H
Acetate	1.79 (s)	3	L, H
Alanine	1.35 (d)	3	L, H
Aspartate	2.69 (dd)	2	L, H
Choline	3.08 (s)	9	L, H
Creatine	2.91 (s)	3	L, H
Creatinine	2.92(s)	3	L
Formate	8.33 (s)	1	L
Fumarate	6.39 (s)	1	L, H
Beta-Glucose	5.11 (d)	1	L, H
Glutamate	2.22 (m)	2	L, H
Glutamine	2.32 (m)	2	L, H
Histidine	7.75 (d)	1	L
Hypoxanthine	8.04 (d)	2	H
Inosine	8.22 (s)	1	L, H
Isoleucine	0.89 (t)	3	L
Lactate	1.20 (d)	3	L, H
Leucine	0.83 (m)	6	L
Methionine	2.49 (t)	2	H
Niacinamide	8.81 (s)	1	H
Phenylalanine	7.20 (d)	2	L
Succinate	2.27 (s)	4	L, H
Taurine	3.12 (m)	2	H
Tyrosine	7.06 (d)	2	L
Valine	0.91 (d)	3	L
–CH=CH– (olefinic acyl bonds)	5.32 (m)	-	L, H, A
Docosahexaenoic Acid	2.36 (d)	4	H
Linoleic Acid	2.73 (t)	2	L, H, A
Monounsaturated Fatty Acids	1.97 (m)	2	L, H, A
Omega-3	0.92 (t)	3	L, H, A
Phosphatidylcholine	3.14 (s)	9	L, H, A
Phosphatidylethanolamine	3.08 (t)	2	L, H, A
Sphingomyelin	3.13 (s)	9	L, H, A
Total Cholesterol	0.63 (s)	3	L, H
Total Fatty Acids	0.83 (m)	3	L, H, A
Triglycerides	4.27 (dd)	2	L, H, A

## Data Availability

The metabolite concentration data are included in the Tables in this article.

## References

[1] Hirode G., Wong R.J. (2020). Trends in the Prevalence of Metabolic Syndrome in the United States, 2011–2016. JAMA.

[2] Saklayen M.G. (2018). The Global Epidemic of the Metabolic Syndrome. Curr. Hypertens. Rep..

[3] Zhou L.Y., Deng M.Q., Zhang Q., Xiao X.H. (2020). Early-life nutrition and metabolic disorders in later life: A new perspective on energy metabolism. Chin. Med. J..

[4] Nilsson P.M., Tuomilehto J., Ryden L. (2019). The metabolic syndrome—What is it and how should it be managed?. Eur. J. Prev. Cardiol..

[5] Wong S.K., Chin K.Y., Suhaimi F.H., Fairus A., Ima-Nirwana S. (2016). Animal models of metabolic syndrome: A review. Nutr. Metab..

[6] Hoyas I., Leon-Sanz M. (2019). Nutritional Challenges in Metabolic Syndrome. J. Clin. Med..

[7] Regmi P., Heilbronn L.K. (2020). Time-Restricted Eating: Benefits, Mechanisms, and Challenges in Translation. iScience.

[8] Rothschild J., Hoddy K.K., Jambazian P., Varady K.A. (2014). Time-restricted feeding and risk of metabolic disease: A review of human and animal studies. Nutr. Rev..

[9] Adafer R., Messaadi W., Meddahi M., Patey A., Haderbache A., Bayen S., Messaadi N. (2020). Food Timing, Circadian Rhythm and Chrononutrition: A Systematic Review of Time-Restricted Eating’s Effects on Human Health. Nutrients.

[10] Che T., Yan C., Tian D., Zhang X., Liu X., Wu Z. (2021). Time-restricted feeding improves blood glucose and insulin sensitivity in overweight patients with type 2 diabetes: A randomised controlled trial. Nutr. Metab..

[11] Greenwell B.J., Trott A.J., Beytebiere J.R., Pao S., Bosley A., Beach E., Finegan P., Hernandez C., Menet J.S. (2019). Rhythmic Food Intake Drives Rhythmic Gene Expression More Potently than the Hepatic Circadian Clock in Mice. Cell. Rep..

[12] Weger B.D., Gobet C., David F.P.A., Atger F., Martin E., Phillips N.E., Charpagne A., Weger M., Naef F., Gachon F. (2021). Systematic analysis of differential rhythmic liver gene expression mediated by the circadian clock and feeding rhythms. Proc. Natl. Acad. Sci. USA.

[13] Świątkiewicz I., Woźniak A., Taub P.R. (2021). Time-Restricted Eating and Metabolic Syndrome: Current Status and Future Perspectives. Nutrients.

[14] Wilkinson M.J., Manoogian E.N.C., Zadourian A., Lo H., Fakhouri S., Shoghi A., Wang X., Fleischer J.G., Navlakha S., Panda S. (2020). Ten-Hour Time-Restricted Eating Reduces Weight, Blood Pressure, and Atherogenic Lipids in Patients with Metabolic Syndrome. Cell. Metab..

[15] Shi D., Chen J., Wang J., Yao J., Huang Y., Zhang G., Bao Z. (2019). Circadian Clock Genes in the Metabolism of Non-alcoholic Fatty Liver Disease. Front. Physiol..

[16] Chait A., den Hartigh L.J. (2020). Adipose Tissue Distribution, Inflammation and Its Metabolic Consequences, Including Diabetes and Cardiovascular Disease. Front. Cardiovasc. Med..

[17] Ferrero K.M., Koch W.J. (2020). Metabolic Crosstalk between the Heart and Fat. Korean Circ. J..

[18] Grundy S.M. (2015). Adipose tissue and metabolic syndrome: Too much, too little or neither. Eur. J. Clin. Investig..

[19] Kim J.B. (2016). Dynamic cross talk between metabolic organs in obesity and metabolic diseases. Exp. Mol. Med..

[20] Nakamura M., Sadoshima J. (2014). Heart over mind: Metabolic control of white adipose tissue and liver. EMBO Mol. Med..

[21] Romero A., Eckel J. (2021). Organ Crosstalk and the Modulation of Insulin Signaling. Cells.

[22] Guasch-Ferre M., Bhupathiraju S.N., Hu F.B. (2018). Use of Metabolomics in Improving Assessment of Dietary Intake. Clin. Chem..

[23] Ryan E.P., Heuberger A.L., Broeckling C.D., Borresen E.C., Tillotson C., Prenni J.E. (2013). Advances in Nutritional Metabolomics. Curr. Metab..

[24] Schmidt J.A., Fensom G.K., Rinaldi S., Scalbert A., Gunter M.J., Holmes M.V., Key T.J., Travis R.C. (2021). NMR Metabolite Profiles in Male Meat-Eaters, Fish-Eaters, Vegetarians and Vegans, and Comparison with MS Metabolite Profiles. Metabolites.

[25] Abbondante S., Eckel-Mahan K.L., Ceglia N.J., Baldi P., Sassone-Corsi P. (2016). Comparative Circadian Metabolomics Reveal Differential Effects of Nutritional Challenge in the Serum and Liver. J. Biol. Chem..

[26] Ye Y., Xu H., Xie Z., Wang L., Sun Y., Yang H., Hu D., Mao Y. (2020). Time-Restricted Feeding Reduces the Detrimental Effects of a High-Fat Diet, Possibly by Modulating the Circadian Rhythm of Hepatic Lipid Metabolism and Gut Microbiota. Front. Nutr..

[27] Li J., Vosegaard T., Guo Z. (2017). Applications of nuclear magnetic resonance in lipid analyses: An emerging powerful tool for lipidomics studies. Prog. Lipid Res..

[28] Edison A.S., Colonna M., Gouveia G.J., Holderman N.R., Judge M.T., Shen X., Zhang S. (2020). NMR: Unique Strengths That Enhance Modern Metabolomics Research. Anal. Chem..

[29] Bharti S.K., Roy R. (2012). Quantitative NMR Spectroscopy. TrAC Trends Anal. Chem..

[30] Hatzakis E. (2019). Nuclear Magnetic Resonance (NMR) Spectroscopy in Food Science: A Comprehensive Review. Compr. Rev. Food Sci. Food Saf..

[31] Krishnamurthy K. (2013). CRAFT (complete reduction to amplitude frequency table)—Robust and time-efficient Bayesian approach for quantitative mixture analysis by NMR. Magn. Reson. Chem..

[32] Johnson H., Puppa M., van der Merwe M., Tipirneni-Sajja A. (2021). CRAFT for NMR lipidomics: Targeting lipid metabolism in leucine-supplemented tumor-bearing mice. Magn. Reson. Chem..

[33] Krishnamurthy K. (2021). Complete Reduction to Amplitude Frequency Table (CRAFT)—A perspective. Magn. Reson. Chem..

[34] Quirk J.D., Bretthorst G.L., Garbow J.R., Ackerman J.J.H. (2019). Magnetic resonance data modeling: The Bayesian analysis toolbox. Concepts Magn. Reson. Part A.

[35] Hatori M., Vollmers C., Zarrinpar A., DiTacchio L., Bushong E.A., Gill S., Leblanc M., Chaix A., Joens M., Fitzpatrick J.A. (2012). Time-restricted feeding without reducing caloric intake prevents metabolic diseases in mice fed a high-fat diet. Cell. Metab..

[36] de Goede P., Foppen E., Ritsema W.I.G.R., Korpel N.L., Yi C.-X., Kalsbeek A. (2019). Time-Restricted Feeding Improves Glucose Tolerance in Rats, but Only When in Line With the Circadian Timing System. Front. Endocrinol..

[37] Olsen M.K., Choi M.H., Kulseng B., Zhao C.-M., Chen D. (2017). Time-restricted feeding on weekdays restricts weight gain: A study using rat models of high-fat diet-induced obesity. Physiol. Behav..

[38] Sun S., Hanzawa F., Kim D., Umeki M., Nakajima S., Sakai K., Ikeda S., Mochizuki S., Oda H. (2019). Circadian rhythm–dependent induction of hepatic lipogenic gene expression in rats fed a high-sucrose diet. J. Biol. Chem..

[39] Chung H., Chou W., Sears D.D., Patterson R.E., Webster N.J., Ellies L.G. (2016). Time-restricted feeding improves insulin resistance and hepatic steatosis in a mouse model of postmenopausal obesity. Metabolism.

[40] Das M., Ellies L.G., Kumar D., Sauceda C., Oberg A., Gross E., Mandt T., Newton I.G., Kaur M., Sears D.D. (2021). Time-restricted feeding normalizes hyperinsulinemia to inhibit breast cancer in obese postmenopausal mouse models. Nat. Commun..

[41] Power M.L., Schulkin J. (2008). Sex differences in fat storage, fat metabolism, and the health risks from obesity: Possible evolutionary origins. Br. J. Nutr..

[42] Ethun K. (2016). Sex and Gender Differences in Body Composition, Lipid Metabolism, and Glucose Regulation. Sex Differences in Physiology.

[43] Schneider J., Han W.H., Matthew R., Sauve Y., Lemieux H. (2020). Age and sex as confounding factors in the relationship between cardiac mitochondrial function and type 2 diabetes in the Nile Grass rat. PLoS ONE.

[44] Subramaniam A., Landstrom M., Luu A., Hayes K.C. (2018). The Nile Rat (Arvicanthis niloticus) as a Superior Carbohydrate-Sensitive Model for Type 2 Diabetes Mellitus (T2DM). Nutrients.

[45] Noda K., Melhorn M.I., Zandi S., Frimmel S., Tayyari F., Hisatomi T., Almulki L., Pronczuk A., Hayes K.C., Hafezi-Moghadam A. (2010). An animal model of spontaneous metabolic syndrome: Nile grass rat. FASEB J..

[46] Ramanathan C., Johnson H., Sharma S., Son W., Puppa M., Rohani S.N., Tipirneni-Sajja A., Bloomer R.J., van der Merwe M. (2022). Early Time-Restricted Feeding Amends Circadian Clock Function and Improves Metabolic Health in Male and Female Nile Grass Rats. Medicines.

[47] Suganami T., Tanaka M., Ogawa Y. (2012). Adipose tissue inflammation and ectopic lipid accumulation. Endocr. J..

[48] Peterson L.R., Herrero P., Schechtman K.B., Racette S.B., Waggoner A.D., Kisrieva-Ware Z., Dence C., Klein S., Marsala J., Meyer T. (2004). Effect of obesity and insulin resistance on myocardial substrate metabolism and efficiency in young women. Circulation.

[49] Kučera O., Garnol T., Lotková H., Staňková P., Mazurová Y., Hroch M., Bolehovská R., Roušar T., Červinková Z. (2011). The Effect of Rat Strain, Diet Composition and Feeding Period on the Development of a Nutritional Model of Non-Alcoholic Fatty Liver Disease in Rats. Physiol. Res..

[50] Guillou H., Yaligar J., Gopalan V., Kiat O.W., Sugii S., Shui G., Lam B.D., Henry C.J., Wenk M.R., Tai E.S. (2014). Evaluation of Dietary Effects on Hepatic Lipids in High Fat and Placebo Diet Fed Rats by In Vivo MRS and LC-MS Techniques. PLoS ONE.

[51] Nagarajan V., Gopalan V., Kaneko M., Angeli V., Gluckman P., Richards A.M., Kuchel P.W., Velan S.S. (2013). Cardiac function and lipid distribution in rats fed a high-fat diet: In vivo magnetic resonance imaging and spectroscopy. Am. J. Physiol. Heart Circ. Physiol..

[52] Harasim E., Stępek T., Konstantynowicz-Nowicka K., Baranowski M., Górski J., Chabowski A. (2015). Myocardial Lipid Profiling During Time Course of High Fat Diet and its Relationship to the Expression of Fatty Acid Transporters. Cell. Physiol. Biochem..

[53] Rangel-Huerta O.D., Pastor-Villaescusa B., Gil A. (2019). Are we close to defining a metabolomic signature of human obesity? A systematic review of metabolomics studies. Metabolomics.

[54] Caputo T., Gilardi F., Desvergne B. (2017). From chronic overnutrition to metaflammation and insulin resistance: Adipose tissue and liver contributions. FEBS Lett..

[55] Chaix A., Zarrinpar A. (2015). The effects of time-restricted feeding on lipid metabolism and adiposity. Adipocyte.

[56] Aguila M.B., Sun S., Hanzawa F., Umeki M., Ikeda S., Mochizuki S., Oda H. (2018). Time-restricted feeding suppresses excess sucrose-induced plasma and liver lipid accumulation in rats. PLoS ONE.

[57] Dyar K.A., Lutter D., Artati A., Ceglia N.J., Liu Y., Armenta D., Jastroch M., Schneider S., de Mateo S., Cervantes M. (2018). Atlas of Circadian Metabolism Reveals System-wide Coordination and Communication between Clocks. Cell.

[58] Kessler K., Pivovarova-Ramich O. (2019). Meal Timing, Aging, and Metabolic Health. Int. J. Mol. Sci..

[59] Wells A., Barrington W.T., Dearth S., May A., Threadgill D.W., Campagna S.R., Voy B.H. (2018). Tissue Level Diet and Sex-by-Diet Interactions Reveal Unique Metabolite and Clustering Profiles Using Untargeted Liquid Chromatography-Mass Spectrometry on Adipose, Skeletal Muscle, and Liver Tissue in C57BL6/J Mice. J. Proteome. Res..

[60] Sivakumar R., Anandh Babu P.V., Shyamaladevi C.S. (2008). Protective effect of aspartate and glutamate on cardiac mitochondrial function during myocardial infarction in experimental rats. Chem. Biol. Interact..

[61] Tang W., Wu J., Jin S., He L., Lin Q., Luo F., He X., Feng Y., He B., Bing P. (2020). Glutamate and aspartate alleviate testicular/epididymal oxidative stress by supporting antioxidant enzymes and immune defense systems in boars. Sci. China Life Sci..

[62] Amiel A., Tremblay-Franco M., Gautier R., Ducheix S., Montagner A., Polizzi A., Debrauwer L., Guillou H., Bertrand-Michel J., Canlet C. (2019). Proton NMR Enables the Absolute Quantification of Aqueous Metabolites and Lipid Classes in Unique Mouse Liver Samples. Metabolites.

[63] Sostare J., Di Guida R., Kirwan J., Chalal K., Palmer E., Dunn W.B., Viant M.R. (2018). Comparison of modified Matyash method to conventional solvent systems for polar metabolite and lipid extractions. Anal. Chim. Acta.

[64] Bernstein H.S., Overmyer K.A., Thonusin C., Qi N.R., Burant C.F., Evans C.R. (2015). Impact of Anesthesia and Euthanasia on Metabolomics of Mammalian Tissues: Studies in a C57BL/6J Mouse Model. PLoS ONE.

[65] Nagana Gowda G.A., Abell L., Lee C.F., Tian R., Raftery D. (2016). Simultaneous Analysis of Major Coenzymes of Cellular Redox Reactions and Energy Using ex Vivo 1H NMR Spectroscopy. Anal. Chem..

[66] Grinde M.T., Giskeødegård G.F., Andreassen T., Tessem M.-B., Bathen T.F., Moestue S.A. (2019). Biomarker Discovery Using NMR-Based Metabolomics of Tissue. NMR-Based Metabolomics.

[67] Leary S., Underwood W., Anthony R., Cartner S., Grandin T., Greenacre C., Gwaltney-Brant S., McCrackin M.A., Meyer R., Miller D. (2020). AVMA Guidelines for the Euthanasia of Animals: 2020 Edition.

[68] Crook A.A., Powers R. (2020). Quantitative NMR-Based Biomedical Metabolomics: Current Status and Applications. Molecules.

[69] Aboualizadeh E., Carmichael O.T., He P., Albarado D.C., Morrison C.D., Hirschmugl C.J. (2017). Quantifying Biochemical Alterations in Brown and Subcutaneous White Adipose Tissues of Mice Using Fourier Transform Infrared Widefield Imaging. Front. Endocrinol..

[70] Wishart D.S., Guo A., Oler E., Wang F., Anjum A., Peters H., Dizon R., Sayeeda Z., Tian S., Lee B.L. (2022). HMDB 5.0: The Human Metabolome Database for 2022. Nucleic Acids Res..

[71] Nagana Gowda G.A., Raftery D. (2019). Analysis of Plasma, Serum, and Whole Blood Metabolites Using (1)H NMR Spectroscopy. Methods Mol. Biol..

[72] Chi Y., Gupta R.K. (1998). Alterations in membrane fatty acid unsaturation and chain length in hypertension as observed by 1H NMR spectroscopy. Am. J. Hypertens.

[73] Hernandez-Baixauli J., Quesada-Vazquez S., Marine-Casado R., Gil Cardoso K., Caimari A., Del Bas J.M., Escote X., Baselga-Escudero L. (2020). Detection of Early Disease Risk Factors Associated with Metabolic Syndrome: A New Era with the NMR Metabolomics Assessment. Nutrients.

[74] Lei S.S., Zhang N.Y., Zhou F.C., He X., Wang H.Y., Li L.Z., Zheng X., Dong Y.J., Luo R., Li B. (2021). Dendrobium officinale Regulates Fatty Acid Metabolism to Ameliorate Liver Lipid Accumulation in NAFLD Mice. Evid. Based Complement Altern. Med..

[75] When It Makes Sense to Not Correct for Multiple Comparisons. https://www.graphpad.com/guides/prism/latest/statistics/stat_when_to_not_correct_for_2.htm.

[76] Rothman K.J. (1990). No adjustments are needed for multiple comparisons. Epidemiology.

[77] Muhammad I.F., Borne Y., Zaigham S., Soderholm M., Johnson L., Persson M., Melander O., Engstrom G. (2021). Comparison of risk factors for ischemic stroke and coronary events in a population-based cohort. BMC Cardiovasc. Disord..

[78] Perneger T.V. (1998). What’s wrong with Bonferroni adjustments. BMJ.

[79] Menyhart O., Weltz B., Gyorffy B. (2021). MultipleTesting.com: A tool for life science researchers for multiple hypothesis testing correction. PLoS ONE.

